# Spin structures of the ground states of four body bound systems with spin 3 cold atoms

**DOI:** 10.1038/s41598-021-97521-y

**Published:** 2021-09-09

**Authors:** Y. M. Liu, C. G. Bao

**Affiliations:** 1 Department of physics, Shaoguan University, Shaoguan, 510205 People’s Republic of China; 2 School of Physics, Sun Yat-Sen University, Guangzhou, 510275 People’s Republic of China

**Keywords:** Bose-Einstein condensates, Electronic properties and materials

## Abstract

We consider the case that four spin-3 atoms are confined in an optical trap. The temperature is so low that the spatial degrees of freedom have been frozen. Exact numerical and analytical solutions for the spin-states have been both obtained. Two kinds of phase-diagrams for the ground states (g.s.) have been plotted. In general, the eigen-states with the total-spin *S* (a good quantum number) can be expanded in terms of a few basis-states $f_{S,i}$. Let $P_{f_{S,i}}^{\lambda }$ be the probability of a pair of spins coupled to $\lambda =0, 2, 4$, and 6 in the $f_{S,i}$ state. Obviously, when the strength $g_{\lambda }$ of the $\lambda$-channel is more negative, the basis-state with the largest $P_{f_{S,i}}^{\lambda }$ would be more preferred by the g.s.. When two strengths are more negative, the two basis-states with the two largest probabilities would be more important components. Thus, based on the probabilities, the spin-structures (described via the basis-states) can be understood. Furthermore, all the details in the phase-diagrams, say, the critical points of transition, can also be explained. Note that, for $f_{S,i}$, $P_{f_{S,i}}^{\lambda }$ is completely determined by symmetry. Thus, symmetry plays a very important role in determining the spin-structure of the g.s..

## Introduction

It is recalled that, due to the realization of optical trapping about 20 years ago, the field of Bose–Einstein condensates has been greatly extended and the spin-degrees of freedom begin to play their roles. On the other hand, a notable progress in recent years is the technique in the trapping and manipulation of a few cold atoms (molecules)^1^. This technique could also extend the field greatly from traditional many-body systems to cold few-body systems. In the theoretical aspect, the former can only be solved approximately, while the latter can be solved exactly and detailed analysis on the spin-structures can be made. Thus the knowledge extracted from few-body systems would be a complement to those from many-body systems. Furthermore, for cold atoms, the temperature can be tuned so low (say, ${\text{ T }}<10^{-10}$ K) that the spatial degrees of freedom are nearly frozen. This leads to a kind of cold few-body systems having only spin-degrees of freedom. Note that all few-body systems are strongly constrained by symmetry so that the quantum states are governed by a few quantum numbers. Obviously, due to the difference in degrees of freedom, the effects of symmetry constraint imposing on usual and cold few-body systems are different (as shown in a previous paper^2^). Thus, the field of the study of few-body systems could also be thereby extended and rich physics would be involved. Therefore, the study of cold few-body systems, they are scarcely studied before, is meaningful.

For many-body systems, there are a number of literatures dedicated to the study of spin-1^3–11^ and spin-2 cold atoms^10,12–19^. Those for spin-3 condensates are fewer, where the spin-structures appear to be complicated^20–26^. This paper, as a continuation of^2^, is dedicated to four-body systems with spin-3 cold atoms. The purpose is to find out the spin-structures of the ground states (g.s.). Note that the interaction contains four parameters $\{g_{\lambda }\}$ (where $\lambda$ is the coupled spin of two atoms). A negative $g_{\lambda }$ would push a pair of atoms to form a [$\lambda$]-pair ($\lambda =0$, 2, 4, and 6). We believe that, when $g_{\lambda }$ is sufficiently negative, the [$\lambda$]-pairs would be important constituents. When two or more $g_{\lambda }$ are negative, there is competition among them. We will see how the competition would be under the constraint from symmetry.

## Spin-dependent Hamiltonian and the eigen-states

Let *N* spin-3 atoms (say, Cr, Mo, Sn, Pu) be confined in an optical trap. It is assumed that the temperature is so low and the binding is so strong that all the particles have condensed to a spatial state $\phi (\mathbf {r})$ which is most favorable for binding. While all the spatial degrees of freedom are frozen, the spin-degrees of freedom remain free, therefore various spin-structures can be formed. These structures depend essentially on the spin-dependent Hamiltonian, which can be written as

$$\begin{aligned} H_{spin}= & {} \sum _{i<j}V_{ij},\\ V_{ij}= & {} \sum _{\lambda }g_{\lambda }P_{\lambda }^{ij}, \end{aligned}$$

where *i* (*j*) denotes a particle. $$\lambda =0$$, 2, 4, and 6 is the coupled spin of a pair, $$P_{\lambda }^{ij}$$ is the projector to the $$\lambda $$ -channel. $$g_{\lambda }$$ is the weighted strength which is a product of the strength and the integral $$\int \phi ^{4}d\mathbf {r}$$. The latter embodies the effect of spatial profile. The dipole–dipole (*d*–*d*) coupling between a pair of atoms is relatively weak (for $$^{52}$$Cr as an example, the strength of the *d*–*d* coupling $$c_{dd}=0.004g_{6}$$), therefore is neglected. In fact, the calculation in^21^ demonstrates that the g.s. of $$^{52}$$Cr does not seem to depend on the *d*–*d* coupling. An important feature of $$ H_{spin}$$ is the conservation of the total spin *S* and its Z-component *M* . Thus the eigen-energies and eigen-states of $$H_{spin}$$ are denoted as $$ E_{SM}$$ and $$\psi _{SM}$$ (the subscript *M* might be neglected).

We introduce the Fock-states $|\alpha \rangle \equiv |N_{3\alpha },N_{2\alpha },\ldots N_{-3,\alpha }\rangle$, where $\alpha$ represents a set of seven numbers $\{N_{\mu \alpha }\}$ ($-3\le \mu \le 3$), $N_{\mu \alpha }$ is the number of particles in $\mu$ magnetic component. Obviously, $\Sigma _{\mu }N_{\mu \alpha }=N$ and $\Sigma _{\mu }\mu N_{\mu \alpha }=M$. The Fock-states are adopted as basis-states for diagonalizing $H_{spin}$. The matrix element is

$$\begin{aligned} \langle \alpha ^{\prime }|H_{spin}|\alpha \rangle= & {} \frac{1}{2}\underset{ \mu ^{\prime }\nu ^{\prime }\mu \nu }{\Sigma }\delta _{\mu ^{\prime }+\nu ^{\prime },\mu +\nu }\sum _{\lambda }g_{\lambda }C_{3\mu ^{\prime };3\nu ^{\prime }}^{\lambda ,\mu ^{\prime }+\nu ^{\prime }}C_{3\mu ;3\nu }^{\lambda ,\mu +\nu }\\&\cdot \left( \overset{\_}{\delta }_{\mu ^{\prime }\nu ^{\prime }}\overset{\_}{\delta } _{\mu \nu }\sqrt{N_{\mu ^{\prime }}^{\prime }N_{\nu ^{\prime }}^{\prime }N_{\mu }N_{\nu }}\delta _{[\alpha ^{\prime }]_{\mu ^{\prime }\nu ^{\prime }};[\alpha ]_{\mu \nu }} \right. \\&\left. +\overset{\_}{\delta }_{\mu ^{\prime }\nu ^{\prime }}\overset{}{\delta } _{\mu \nu }\sqrt{N_{\mu ^{\prime }}^{\prime }N_{\nu ^{\prime }}^{\prime }N_{\mu }(N_{\mu }-1)}\delta _{[\alpha ^{\prime }]_{\mu ^{\prime }\nu ^{\prime }};[\alpha ]_{\mu \mu }} \right. \\&\left. +\overset{}{\delta }_{\mu ^{\prime }\nu ^{\prime }}\overset{\_}{\delta } _{\mu \nu }\sqrt{N_{\mu ^{\prime }}^{\prime }(N_{\mu ^{\prime }}^{\prime }-1)N_{\mu }N_{\nu }}\delta _{[\alpha ^{\prime }]_{\mu ^{\prime }\mu ^{\prime }};[\alpha ]_{\mu \nu }} \right. \\&\left. +\overset{}{\delta }_{\mu ^{\prime }\nu ^{\prime }}\overset{}{\delta } _{\mu \nu }\sqrt{N_{\mu ^{\prime }}^{\prime }(N_{\mu ^{\prime }}^{\prime }-1)N_{\mu }(N_{\mu }-1)}\delta _{[\alpha ^{\prime }]_{\mu ^{\prime }\mu ^{\prime }};[\alpha ]_{\mu \mu }} \right) \ \ \end{aligned}$$

where $|\alpha ^{\prime }\rangle \equiv |N_{3\alpha }^{\prime },\cdot \cdot \cdot \rangle$, $\delta _{\mu \nu }=1$ or 0 (if $\mu =\nu$ or $\ne \nu$ ), $\overset{\_}{\delta }_{\mu \nu }=1-\delta _{\mu \nu }$, $\delta _{[\beta ];[\alpha ]}=1$ (if all the seven numbers in $[\beta ]$ are one-to-one identical to those in $[\alpha ]$) or 0 (otherwise), the Clebsch–Gordan coefficients have been introduced. Carrying out the diagonalization, $E_{SM}$ together with

$$\begin{aligned} \psi _{SM}=\underset{\alpha }{\sum }D_{\alpha }^{S}|\alpha \rangle \end{aligned}$$

can be obtained. The total number of Fock-states is bound by *N* and *M*. Since no magnetic field is applied, *S* of an eigen-state can be known by its degeneracy. In particular, the lowest eigen-state (g.s.) is denoted as $\Psi _{S(gs)}$ which we will focus on.

## Spin-structures based on the pairs

**Figure 1:**
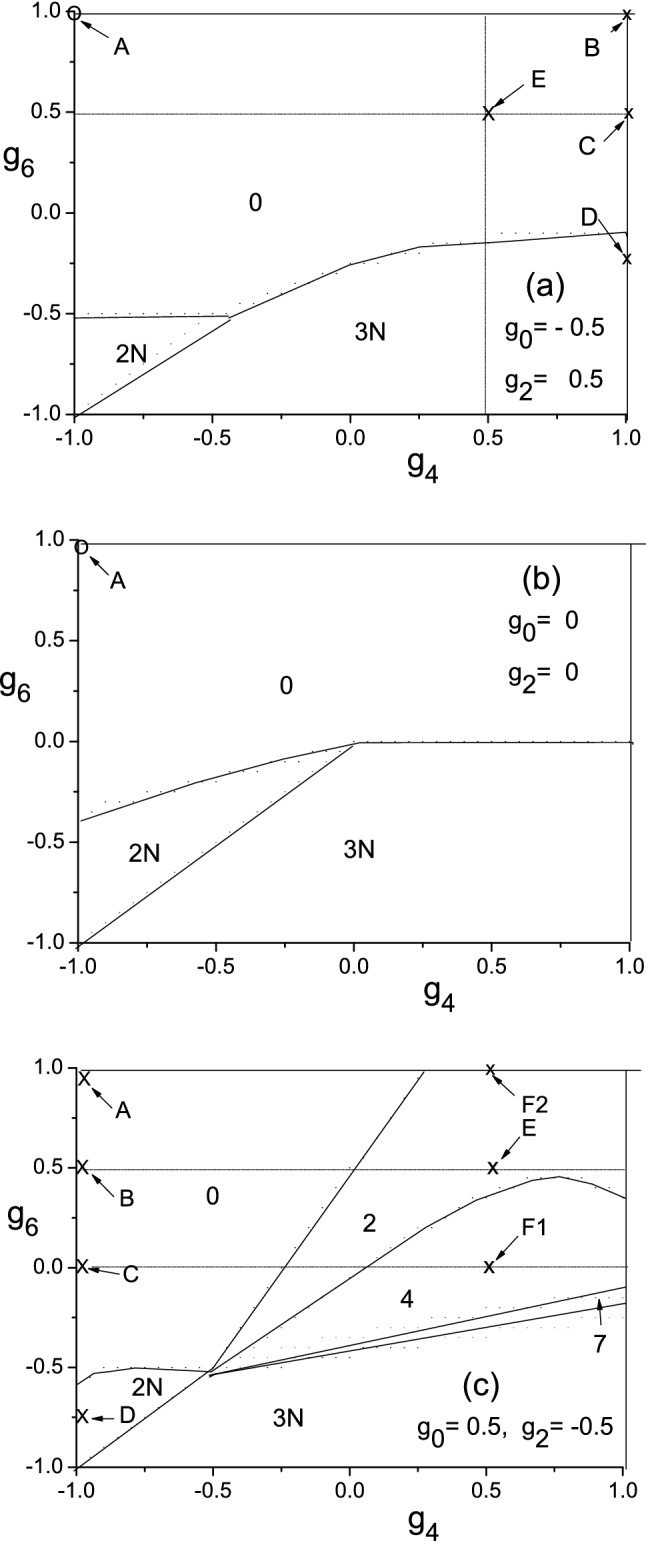
Phase diagrams of the g.s. of N = 4 systems against $g_{4}$ and $g_{6}$, while $g_{0}$ and $g_{2}$ are fixed and marked in the panels. *S* is marked on the associated zone.

After the diagonalization of $H_{spin}$, the parameter space can be divided into zones according to *S*, and the phase diagram thereby can be plotted. To reduce the complexity, we use three 2-dimensional subspaces to replace the 4-dimensional parameter space as shown in Fig. 1. In each of these subspaces $g_{4}$ and $g_{6}$ are variable, while $g_{0}$ and $g_{2}$ are fixed. There are three possible cases (1) $g_{0}<g_{2}$, (2) $g_{0}\simeq g_{2}$, and (3) $g_{0}>g_{2}$. Note that the spin-structures will neither be changed when all the $\{g_{\lambda }\}$ are shifted with the same value, nor when the unit for $\{g_{\lambda }\}$ is changed. For case (1), let the set $\{g_{\lambda }\}$ be shifted so that $(g_{0}+g_{2})/2=0$, then a unit is adopted so that $g_{0}=-0.5$ and $g_{2}=0.5$ (Fig. 1a). For case (2), as an approximation, we assume $g_{0}=g_{2}$. Then, $\{g_{\lambda }\}$ is shifted so that $g_{0}=g_{2}=0$ (Fig. 1b). For case (3), similarly, we have $g_{0}=0.5$ and $g_{2}=-0.5$ (Fig. 1c). For all the three cases, the ranges of $g_{4}$ and $g_{6}$ are from $-1$ to $+1$. In the qualitative sense, the feature of a 4-dimensional diagram can be roughly illustrated via these three 2-dimensional diagrams.

To understand better the underlying physics, in addition to numerical solutions, we look for analytical solutions. Letbe a basis-state,where $\mathfrak {S}$ is an operator for symmetrization and normalization, $\chi (i)$ is the spin-state of the i-th particle, particles 1 and 2 (3 and 4) are coupled to $\lambda _{a}$ ($\lambda _{b}$), $\lambda _{a}$ and $\lambda _{b}$ should be even and coupled to *S*. Note that $\overset{}{ \varphi }_{S;\lambda _{a}\lambda _{b}}$ has not yet been symmetrized, but  is. When *S* is fixed while $\lambda _{a}$ and $\lambda _{b}$ are variable, the set  can also be used as (non-orthogonal) basis-states for $\psi _{SM}$. It turns out that, for N = 4, the multiplicity of every $\psi _{SM}$ is very small ($\le 3$ ). Thus $H_{spin}$ can be analytically diagonalized. Examples are given below.

By recoupling the spins, we havewhere

$$\begin{aligned} C_{S;\lambda _{a}\lambda _{b};\lambda _{a}^{\prime }\lambda _{b}^{\prime }}=\gamma \left( \delta _{\lambda _{a}\lambda _{a}^{\prime }}\delta _{\lambda _{b}\lambda _{b}^{\prime }}+(-1)^{S}\delta _{\lambda _{a}\lambda _{b}^{\prime }}\delta _{\lambda _{b}\lambda _{a}^{\prime }}+4\overset{\_\_}{ \lambda _{a}}\overset{\_\_}{\lambda _{b}}\overset{\_\_}{\lambda _{a}^{\prime }}\overset{\_\_}{\lambda _{b}^{\prime }} \begin{Bmatrix} 3&3&\lambda _{a} \\ 3&3&\lambda _{b} \\ \lambda _{a}^{\prime }&\lambda _{b}^{\prime }&S \end{Bmatrix} \right) \ \ \end{aligned}$$

where $\gamma$ is a coefficient for normalization, the quantity with {} is a 9-j symbol, and $\overset{\_\_}{\lambda _{a}}\equiv \sqrt{2\lambda _{a}+1}$, etc.

The multiplicity of $S=0$ states is two. Therefore, among the four basis-states , it is sufficient to choose  and  for the expansion of $\psi _{SM}$. Other  state is simply a linear combination of them. Note that these two basis-states are not exactly orthogonal to each other. Instead, , where $C_{0;\lambda \lambda ;\lambda ^{\prime }\lambda ^{\prime }}$ is given in the table.For $S=0$ states, the associated matrix elements arewhere the set $\{C_{0;\lambda ^{\prime }\lambda ^{\prime };\lambda \lambda }\}$ are listed in the table:

**Table 1:** The coefficients in the expansion of $\psi _{SM}$ when $S=0$

$\lambda$	0	2	4	6
$C_{0;00;\lambda \lambda }$	0.6547	0.3253	0.4364	0.5245
$C_{0;22;\lambda \lambda }$	0.3061	0.6958	$-0.4591$	0.4598
$C_{0;44;\lambda \lambda }$	0.3292	$-0.3681$	0.8679	0.0540
$C_{0;66;\lambda \lambda }$	0.5941	0.5535	0.0810	0.5780

The eigen-energy $E_{0}$ is the root of a two-dimensional homogeneous linear equation,

$$\begin{aligned} \left( E_{0}-H_{4,4}\right) \left( E_{0}-H_{6,6}\right) -\left( E_{0}O_{4,6}-H_{4,6}\right) \left( E_{0}O_{4,6}-H_{4,6}\right) =0 \end{aligned}$$

Making use of Table 1, the eigen-energy of the lower $S=0$ states is

$$\begin{aligned} E_{0(-)}=\frac{6}{1.9826}\left( B-\sqrt{B^{2}-3.9652D}\right) \end{aligned}$$

where

$$\begin{aligned} B=0.4248g_{0}+0.4799g_{2}+0.7467g_{4}+0.3312g_{6} \end{aligned}$$

$$\begin{aligned} D=0.1607g_{0}g_{2}+0.2391g_{0}g_{4}+0.0250g_{0}g_{6} \ \ +0.2603g_{2}g_{4}+0.0589g_{2}g_{6}+0.2473g_{4}g_{6} \end{aligned}$$

The normalized spin-state of the lower $S=0$ state iswhere

$$a_{4}=1/\sqrt{1+x^{2}+2xO_{4,6}}$$, $$ x=(H_{4,4}-E_{0(-)})/(O_{4,6}E_{0(-)}-H_{4,6})$$, and $$a_{6}=xa_{4}$$.

The weight of  in $$\psi _{0(-)}$$ is . Similarly, the weight of  is equal to $$ (a_{6}+a_{4}O_{4,6})^{2}$$. If other  are chosen to replace  and/or  , the resultant $$E_{0(-)}$$ and $$\psi _{0(-)}$$ are not changed.

For $S=2$ and 8 (both have multiplicity two), $E_{S(-)}$ and $\psi _{S(-)}$ can be similarly obtained. For $S=4$ and 6 (both have multiplicity three), the analytical solutions are more complicated.

Whereas for $$S=3$$, 5, 7, 9, 10 and 12 states, all of them have multiplicity one, thus the eigen-state is just , where $$\lambda _{a}$$ and $$ \lambda _{b}$$ are arbitrary even numbers adapted to *S*. For example, when $$S=7$$ we choose $$\lambda _{a}=6$$ and $$\lambda _{b}=4$$, then we havewhere $$C_{7;6,4;2,6}=.6362$$, $$C_{7;6,4;4,6}=.3086$$, $$ C_{7;6,4;6,2}=-C_{7;6,4;2,6}$$, $$C_{7;6,4;6,4}=-C_{7;6,4;4,6}$$, otherwise $$ C_{7;6,4;\lambda _{a}^{\prime }\lambda _{b}^{\prime }}=0$$. The eigen-energy

$$\begin{aligned} E_{S=7}=6\left[ C_{7;6,4;2,6}^{2}g_{2}+C_{7;6,4;4,6}^{2}g_{4}+\left( C_{7;6,4;2,6}^{2} +C_{7;6,4;4,6}^{2}\right) g_{6}\right] \end{aligned}$$

The eigen-energies of other *S*-states with multiplicity can be similarly obtained.It is emphasized that, when other $\lambda _{a}$ and $\lambda _{b}$ are chosen, both $\psi _{7M}$ and $E_{S=7}$ remain the same. These states are strictly determined by symmetry. In particular, when $S=3N$, we have $E_{S=3N}=\frac{N(N-1)}{2}g_{6}$. With the help of the analytical solutions, the physics inherent in Fig. 1 can be better understood, we found

1. When $g_{4}<0$ and $g_{6}>0$ (up-left quadrant in Fig. 1) only $S=0$ state is found.
  Note that, when a $g_{\lambda }$ is more negative than the others, two spins in the g.s. will prefer to be coupled to $\lambda$ and form a [$\lambda$]-pair. Let $\langle (\lambda _{a}\lambda _{b})_{S}|gs\rangle$ be a shortened label for the overlap . We found that, at the point A (where $g_{4}=-1$ and $g_{6}=1$) marked in Fig. 1a–c, $\langle (4,4)_{0}|gs\rangle =0.9883$, 0.9996, and 0.9794, respectively. It implies that the g.s. is essentially composed of two [4]-pairs (due to the very negative $g_{4}$), and they are further coupled to zero, namely, they are lying opposite to each other (due to the very positive $g_{6}$). Besides, at C, B and A marked in Fig.1c (where $g_{6}=0$, 0.5, and 1), $\langle (4,4)_{0}|gs\rangle =$0.9703, 0.9765, and 0.9883, respectively. It implies that, when $g_{6}$ increases from 0, the structure (4,4)$_{0}$ will be more dominant.
2. When $g_{4}$ and $g_{6}$ are both negative (down-left quadrant), $S=0$ , 8, and 12 states are found.
  For Fig. 1c as an example, when $g_{4}=-1$ and $g_{6}=0$ (point C), $-0.75$ (point D), and $-1.1$, we found $\langle (4,4)_{0}|gs\rangle =$0.9703, $\langle (4,4)_{8}|gs\rangle =0.966$, and $\langle (6,6)_{12}|gs\rangle =1$, respectively. Thus *S* undergoes a transition as 0$\rightarrow$8$\rightarrow$12. It implies that the decrease of $g_{6}$ causes first a change of the relative orientation of the two [4]-pairs (from being anti-parallel to parallel), then a succeeded breakdown of the [4]-pairs and leading to a full polarization. The transition of *S* takes place when either $E_{0(gs)}=E_{8(gs)}$ or $E_{8(gs)}=E_{12(gs)}$. Since the analytical expressions of the energies have been given, the critical points of transition can be analytically obtained.
3. In up-right quadrant with $g_{4}>0$ and $g_{6}>0$, if $g_{0}\le 0$ (Fig. 1a,b), we found the [0]-pairs. For example,s at the point B, C and E in Fig. 1a, we have $\langle (0,0)_{0}|gs\rangle =0.982$, 0.970, and 0.993, respectively. Whereas if $g_{2}\le 0$ (1c) we found the [2]-pairs. For examples, at the point F2, E and F1, we have $\langle (2,2)_{2}|gs\rangle =1.000$, $\langle (2,2)_{2}|gs\rangle =0.993$, and $\langle (2,2)_{4}|gs\rangle =$ 0.974, respectively. In these examples we see once more how the relative orientation of the two [$\lambda$]-pairs is adjusted by $g_{6}$.
4. When $g_{4}>0$ and $g_{6}<0$ (down-right quadrant), the g.s. mostly has $S=12$ and the g.s. is fully polarized.
5. Making use of the analytical solutions, all the boundaries in the phase diagrams can be analytically described via the equation $E_{S(gs)}=E_{S^{ \prime }(gs)}$. For an example, in Fig. 1c, the boundary separating the zones with $S=12$ and $S^{\prime }=7$ satisfies $g_{6}=0.1904,g_{4}=0.4048$. This explains why this boundary is a straight line up-rising slowly with $g_{4}$.

## Competition in the formation of pairs

**Figure 2:**
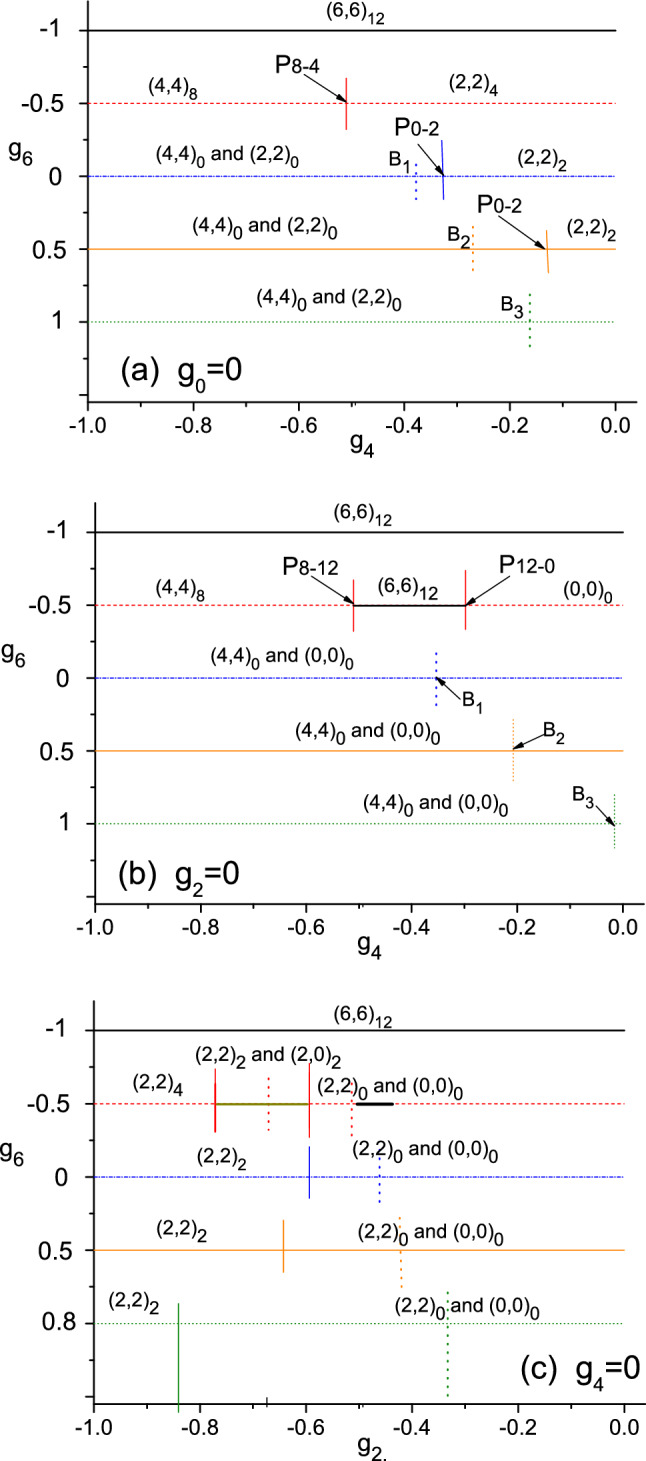
The dominant component(s) in the spin-structure of the g.s. The label of the component $$(\lambda _{a},\lambda _{b})_{S}$$ is marked above the horizontal lines, each is for a given $$g_{6}$$ marked at the left end of the line. For 2a, $$g_{0}=0$$, the abscissa is for $$g_{4}$$, and $$g_{2}=-1-g_{4} $$. For 2b, $$g_{2}=0$$, the abscissa is also for $$g_{4}$$, and $$g_{0}=-1-g_{4}$$. For 2c, $$g_{4}=0$$, the abscissa is for $$g_{2}$$, and $$ g_{0}=-1-g_{2}$$.

From the above section we know that, when a $g_{\lambda }$ is more negative than the others, the [$\lambda$]-pairs will be important. The relative orientation of the spins of pairs depends on $g_{6}$ and will be changed from being anti-parallel to parallel. It is expected that, when $g_{\lambda }$ and $g_{\lambda ^{\prime }}$ are both more negative, there would be a competition between the [$\lambda$]- and [$\lambda ^{\prime }$]-pairs. To clarify, we introduce another kind of phase diagrams as shown in Fig. 2.

In Fig. 2a both $g_{4}$ and $g_{2}$ are negative, thus there is a competition between the [4]- and [2]-pairs

When $g_{6}=-1$ (horizontal black line), due to the strong attraction caused by $g_{6}$, no [$\lambda$]-pairs ($\lambda \ne 6$) would emerge. Instead, all the spins are aligned and the g.s. is fully polarized.

When $$g_{6}=-0.5$$ (dash), both [4]-, [2]-, and [6]-pairs might emerge. When $$ g_{4}<g_{2}$$, the g.s. is dominated by (4,4)$$_{8}$$ (say, when $$g_{4}=-0.75$$, $$\langle (4,4)_{8}|gs\rangle _{g_{4}=-0.75} =0.966$$). There is a critical point p$$_{8\rightarrow 4}$$ located at $$g_{4}=g_{2}=-0.5$$, at which *S* transits from 8 to 4. Afterward, when $$ g_{4}$$ $$(g_{2})$$ increases (decreases) further so that $$g_{2}<g_{4}$$, the g.s. is dominated by (2,2)$$_{4}$$ (say, $$\langle (2,2)_{4}|gs\rangle _{g_{4}=-0.25}=0.967$$). Thus the negative $$g_{6}=-0.5$$ is not sufficient to form the [6]-pairs, but sufficient to bring the spins of the two [4]-pairs or the two [2]-pairs to be parallel.

When $g_{6}=0$ (dash–dot). Due to the vanish of the attraction from $g_{6}$, the two [4]- or [2]-pairs are no more parallel. There is a critical point p$_{0\rightarrow 2}$ (where $g_{4}=-0.333$) and a point of balance B$_{1}$ (where $g_{4}=-0.391$ ). When $g_{4}<{\text{ p }}_{0\rightarrow 2}$, the g.s. is composed of (4,4)$_{0}$ and (2,2)$_{0}$. The pair (4,4)$_{0}$ would be more important if $g_{4}$ < B$_{1}$, whereas (2,2)$_{0}$ would be if $g_{4}$ > B$_{1}$, and they would arrive at a balance at B$_{1}$, i.e., $\langle (4,4)_{0}|gs\rangle _{g_{4}=-0.391}=\langle (2,2)_{0}|gs\rangle _{g_{4}=-0.391}=0.874$ (note: $\langle (4,4)_{0}|(2,2)_{0}\rangle \ne 0$). The point p$_{0\rightarrow 2}$ marks the transition of *S* from 0 to 2. When $g_{4}>{\text{ p }}_{0-2}$, the g.s. is essentially (2,2)$_{2}$ (say, $\langle (2,2)_{2}|gs\rangle _{g_{4}=-0.32}=0.986$, $\langle (2,2)_{2}|gs\rangle _{g_{4}=-0.1}=1.000$).

The case with $g_{6}=0.5$ (solid line in orange) is similar to the case with $g_{6}=0$, except p$_{0\rightarrow 2}=-0.163$, and the balance point B$_{2} =-0.274$. Thus, both p$_{0\rightarrow 2}$ and B$_{2}$ shift to the right.

When $g_{6}=1$ (dot), the case is also similar to the above case, however the critical point shifts to the right and beyond the range $-1{<} g_{4}\le 0$ (therefore it can not be seen). The repulsion caused by $g_{6}$ is sufficiently strong so that the pairs kept to be anti-parallel, and therefore the g.s. is composed of $(4,4)_{0}$ and $(2,2)_{0}$ with the balance point B$_{3}$ at $g_{4}=-0.157$. Say, $\langle (4,4)_{0}|gs\rangle _{g_{4}=-1}=1.000$, $\langle (4,4)_{0}|gs\rangle _{g_{4}=-0.157}=\langle (2,2)_{0}|gs\rangle _{g_{4}=-0.157}=0.874$, and $\langle (2,2)_{0}|gs\rangle _{g_{4}=0}=0.942$.

We found B$_{1}< {\text{ B }}_{2}< {\text{ B }}_{3}$. Note that, when $g_{6}$ is positive, the formation of [6]-pairs is unfavorable to the energy. Let the probability of two spins coupled to $\lambda$ in a state $\Phi$ be $\ P_{\Phi }^{\lambda }$. Then, $P_{(\lambda \lambda )_{0}}^{6}=(C_{0;\lambda \lambda ;6,6})^{2}=(0.4598)^{2}$ (if $\lambda =2$) and $=(0.0540)^{2}$ (if $\lambda =4$). Thus the appearance of the [6]-pairs in (4,4)$_{0}$ is much less probable than in (2,2)$_{0}$. Therefore, the structure (4,4)$_{0}$ would be more favorable than (2,2)$_{0}$ when $g_{6}$ becomes more positive. This explains the reason that the balance point shifts to the right.

Furthermore, when $g_{6}$ increases, the critical point also shifts to the right. This is due to a similar reason that the appearance of the [6]-pairs in (2,2)$_{0}$ is less probable than in (2,2)$_{2}$.

In Fig. 2b both $g_{0}$ and $g_{4}$ are negative.

Figure 2b is comparable with Fig. 2a, but the following distinctions are noticeable.

1. In this case the [0]-pairs and [4]-pairs are competing. Accordingly, when $g_{6}\ge 0$, the two important and competing component are (4,4)$_{0}$ and (0,0)$_{0}$ (rather than (2,2)$_{0}$).
2. When $$g_{6}\ge 0$$, $$g_{0}$$ and $$g_{4}$$ are both negative. Thus, both the [4]-pair and [2]-pair are important and they are competing. Meanwhile, $$ g_{6}$$ is sufficiently positive to keep the two [$$\lambda $$]-pairs anti-parallel so that *S* is kept to be zero and the transition of *S* from 0 to 2 does not appear.
3. When $$g_{6}$$ becomes negative, there is competition among the [6], [4], and [0]-pairs. Say, when $$ g_{6}=-0.5$$ and $$-0.51<g_{4}<-0.29$$, the [6]-pairs emerge in the middle segment of the dash-line. They will be changed to the [4]-pairs if $$g_{4}$$ becomes more negative, or to the [0]-pairs if $$g_{0}$$ becomes more negative. For the dash-line, due to the negative $$g_{6}$$, either the two [4]-pairs or the two [6]-pairs are parallel to each other. This leads to the transition of *S* as $$8\rightarrow 12\rightarrow 0$$ when $$g_{4}$$ increases ($$g_{0}$$ decreases).
4. The shift of the balance point to the right appears again (i.e., B$_{1} {<} {\text{ B }} _{2}< {\text{ B }}_{3}$). Note that $C_{0;0,0;6,6}=0.5245$. Thus the appearance of the [6]-pairs in (4,4)$_{0}$ is also much less probable than in (0,0)$_{0}$. This causes the shift as before.

In Fig. 2c both $g_{0}$ and $g_{2}$ are negative.

When $$g_{6}=-1$$, the g.s. is fully polarized as before. Otherwise, the g.s. is essentially composed of $$(2,2)_{\lambda _{b}}$$ and $$(0,0)_{0}$$ (where $$ \lambda _{b}=4$$, 2, and 0). When $$g_{6}=-0.5$$ we see a chain of transitions: *S*=$$4\rightarrow 2\rightarrow 0\rightarrow 12\rightarrow 0$$.When $$g_{0}$$, $$g_{2}$$ and $$ g_{6}$$ are all close to $$-0.5$$, there is a small segment in bold black line where (6,6)$$ _{12}$$ emerges (similar to the case in Fig. 2b). When $$g_{6}=0,$$ 0.5, and 0.8 (dotted line), we see the transition of $$S=2\rightarrow 0$$. Where the critical point shifts to the left when $$g_{6}$$ increases. It implies that the appearance of the [6]-pairs in (2,2)$$_{0}$$ is less probable than in (2,2)$$ _{2}$$. Whereas the balance point shifts to the right when $$g_{6}$$ increases. It implies that the appearance of the [6]-pairs in (2,2)$$_{0}$$ is less probable than in (0,0)$$_{0}$$.

## Final remarks

The spin-structures of $N=4$ condensates have been studied, both numerical and analytical solutions have been obtained. Thereby two kinds of phase-diagrams for the g.s. have been plotted and explained. From dynamical aspect, the [$\lambda$]-pairs would be important constituents when $g_{\lambda }$ is more negative. However, the probability of the appearance of a [$\lambda$]-pair in a particular component $(\lambda _{a}\lambda _{b})_{S}$ is determined by symmetry. Thus the structure of the g.s. depends not only on the strengths but also on the symmetry constraint. We have calculated the probabilities $P_{(\lambda _{a}\lambda _{b})_{S}}^{\lambda }$ for finding out the important components. The importance is further confirmed by the calculation of the amplitudes $\langle (\lambda _{a}\lambda _{b})_{S}|\Psi _{S(gs)}\rangle$. Obviously, for cold few-body systems, the very small multiplicity of a state is a remarkable feature, thereby the states are tightly (or even completely) constrained by symmetry.

When two or more $g_{\lambda }$ are negative and close to each other, there is competition between various [$\lambda$]-pairs and the most important pair is thereby determined. Note that the magnitude of *S* depends on the relative orientation of the pair-spins $\lambda _{a}$ and $\lambda _{b}$ (if they are nonzero), while the orientation is determined by the strengths. In particular, the sign of $g_{6}$ is crucial which determines whether the two pair-spins are parallel or anti-parallel. Thus the variation of $\{g_{\lambda }\}$ will cause the change of the most important pair and the relative orientation of the pair-spins. This leads to the shift of the balance point and the critical point. The chain of transitions is thereby explained.

The approach of this paper can be generalized to systems with a larger *N*. When *N* is larger, if $g_{\lambda }$ is more negative, the [$\lambda$]-pairs would also be more important in the g.s. There would also be competitions among various [$\lambda$]-pairs. The study of the probability $P_{\Phi }^{\lambda }$ where $\Phi$ is an assumed basis-state would also be helpful for finding out the important component(s) and their alternation. In particular, some very stable spin-structures found in few-body systems could be building blocks for large *N* systems. This is a point to be clarified.
